# OCT Based Interpretation of the Optic Nerve Head Anatomy and Prevalence of Optic Disc Drusen in Patients with Idiopathic Intracranial Hypertension (IIH)

**DOI:** 10.3390/life11060584

**Published:** 2021-06-19

**Authors:** Elisabeth Arnberg Wibroe, Lasse Malmqvist, Steffen Hamann

**Affiliations:** 1 Department of Ophthalmology, Rigshospitalet, 2600 Glostrup, Denmark; lasse.malmqvist@regionh.dk (L.M.); steffen.ellitsgaard.hamann@regionh.dk (S.H.); 2 Department of Clinical Medicine, University of Copenhagen, 2200 Copenhagen N, Denmark

**Keywords:** idiopathic intracranial hypertension, IIH, benign intracranial hypertension, optic disc drusen, ODD, hyperreflective lines, peripapillary ovoid mass-like structures, PHOMS, optical coherence tomography, OCT

## Abstract

We aimed to systematically examine the optic nerve head anatomy in patients with idiopathic intracranial hypertension (IIH) using a standardized optical coherence tomography (OCT) protocol. The study retrospectively included 32 patients diagnosed from 2014 to 2021 with IIH. Using OCT, in accordance with a standardized scanning protocol for patients with optic disc drusen, the presence of optic disc drusen, prelaminar hyperreflective lines, peripapillary hyperreflective ovoid mass-like structures, the retinal nerve fiber layer thickness, and macular ganglion cell layer volume was obtained. Optic disc drusen were found in 3.1%, hyperreflective lines in 31.3%, and peripapillary hyperreflective ovoid mass-like structures in 81.3% of all IIH patients at least three months after the time of diagnosis. We found no significant differences in retinal nerve fiber layer thickness or macular ganglion cell layer volume in patients with hyperreflective lines or PHOMS respectively compared to patients without hyperreflective lines (*p =* 0.1285 and *p* = 0.1835). In conclusion, the prevalence of optic disc drusen in IIH patients is similar to the reported prevalence in the general population. The high prevalence of hyperreflective lines and peripapillary hyperreflective ovoid mass-like structures in IIH patients suggest these structures be a result of crowding in the optic nerve head caused by papilledema.

## 1. Introduction

Idiopathic intracranial hypertension (IIH) is an acquired condition of raised intracranial pressure (ICP) for unknown reasons, which leads to papilledema and subsequent axonal damage [1,2,3]. The incidence is rising rapidly and is reported to be around 0.03–4.69 per 100,000 [4,5]. The risk of acquiring the disease is 4–20 times higher for overweight women in the fertile age and the rising incidence significantly correlates with rising BMI in both genders [4,6,7,8]. IIH is also associated with social deprivation and adverse obstetric outcomes, but IIH can develop independently of gender, age, weight, and socioeconomic status [4,6,9]. Disease duration is months to years, and recurrence of the condition has been shown in up to 28% of the patients [6,10]. The main symptom is a headache and other symptoms might be pulsatile tinnitus, transient visual obscurations, blurred vision, double vision, dizziness, nausea, and cognitive dysfunction [11,12,13]. Signs of IIH can be papilledema and 6th nerve palsy [10].

Papilledema in IIH, and in other conditions of raised ICP, is regularly evaluated using the Frisén and Modified Frisén Scale [14,15]. Although this scale is well suited to ascertain the severity of disc edema in an acute setting, it is based on evaluation of the funduscopic appearance of the disc, and the grading does not tell you anything about the underlying anatomy of the prelaminar optic nerve head. In recent years, the advancement of high-resolution, enhanced depth imaging optical coherence tomography (EDI-OCT) has allowed us to gain more insights into the anatomy of this region of the optic nerve. In EDI-OCT the objective lens is placed closer to the eye providing deeper images of the retina or optic nerve head. Compared with traditional OCT (used for years), this imaging modality can display choroidal structure, scleral canal, and to some extent the optic nerve more clearly. Application in macular studies is discussed by Wang et al. [16]. Three anatomical structures have been identified on EDI-OCT of the optic nerve head: Optic disc drusen (ODD), prelaminar hyperreflective lines (HL), and peripapillary hyper-reflective ovoid mass-like structures (PHOMS). The present study is the first to systematically investigate HL in patients with resolved papilledema. HL or ODD localized deep in the scleral canal can be detected on EDI-OCT but would not necessarily be detected on normal Spectral-domain (SD)-OCT. HL and PHOMS are rather newly described findings on OCT. They are a topic of discussion, and the genesis and impact of HL are still to be fully understood. The IIH Treatment Trail investigated structural findings in SD-OCT in IIH patients [17]. However, they did not use EDI-OCT and did not investigate ODD, HL, and PHOMS.

ODD are calcified deposits present from early childhood in the optic nerve head of about 2% of the background population [18]. ODD are slowly expanding in time and subsequently causing axonal damage [19]. There’s no known treatment and if one has ODD, they do not disappear. Thus, ODD can be diagnosed years after they develop. If the prevalence of ODD in IIH patients is in fact increased, it would be detectable even in people who previously had IIH but do not have signs or symptoms of the disease anymore. In a study from 2016, the prevalence of ODD in patients with resolved papilledema from IIH has been reported as high as 19% [20]. HL are often seen on optical coherence tomography (OCT) in eyes with ODD or in the corresponding eye [18]. They might be early ODD changes. It is a challenge to differentiate between HL representing such ODD precursors and reflections of the lamina cribosa as these structures can appear very similar on OCT [21]. Although HL are probably calcified, they are hidden from ultrasound because of their deep location. PHOMS is a common but nonspecific OCT marker of axoplasmic stasis in the optic nerve head [22]. PHOMS are not themselves ODD or ODD precursors, although they can be seen in association with ODD and a wide spectrum of other conditions including IIH. They are often seen in papilledema [22]. The prevalence of HL or PHOMS in resolved papilledema from IIH has not previously been reported.

The aim of the present study was to use EDI-OCT to systematically investigate the anatomy of the optic nerve head of patients with IIH with a focus on the prevalence of ODD, HL, and PHOMS.

## 2. Materials and Methods

The study is retrospective. Patients who were diagnosed with IIH at Rigshospitalet, Copenhagen, between 2014 and 2021, were included if they had had an EDI-OCT (Spectralis HRA + OCT, Heidelberg Engineering, Heidelberg, Germany) of the optic nerve head performed according to the Optic Disc Drusen Studies Consortium scanning protocol for ODD patients (ODD protocol, see below) [21]. Patients were identified by searching the hospital digital medical journals for the diagnosis of IIH using the diagnostic code for IIH, “benign intracranial hypertension”, dg972. We wanted to ensure that no large disc edema would hide any deep structures and anticipated resolved edema after at least 3 months. Thus, patients with available dense optic nerve head EDI-OCT at the follow-up 3 months or more after IIH diagnosis were identified. Medical records were reviewed to validate the diagnosis and to collect additional information: Clinical data, retinal nerve fiber layer (RNFL) thickness, and ganglion cell layer (GCL) volume. Information on RNFL and GCL was obtained from OCTs performed at the same date and at a time when the patient was considered stable: (1) off medication and/or (2) clinical examination including thorough eye examination and autoperimetry (Octopus) considered normal or (3) after 3 months with unchanged clinical findings.

The ODD protocol includes (1) A dense ONH scan of 97 B-scans (30 µm scan intervals, EDI-OCT), averaging 30 B-scans for identification and quantification of ODD, (2) a 20° six-line radial ONH scan for assessment of scleral canal size, (3) a 12° peripapillary scan for evaluation of RNFL, and (4) a macula scan for evaluation of macular GCL volume. All scans were performed in high-resolution mode with B-scan averaging using the built-in eye-tracking technology.

**Figure 1:**
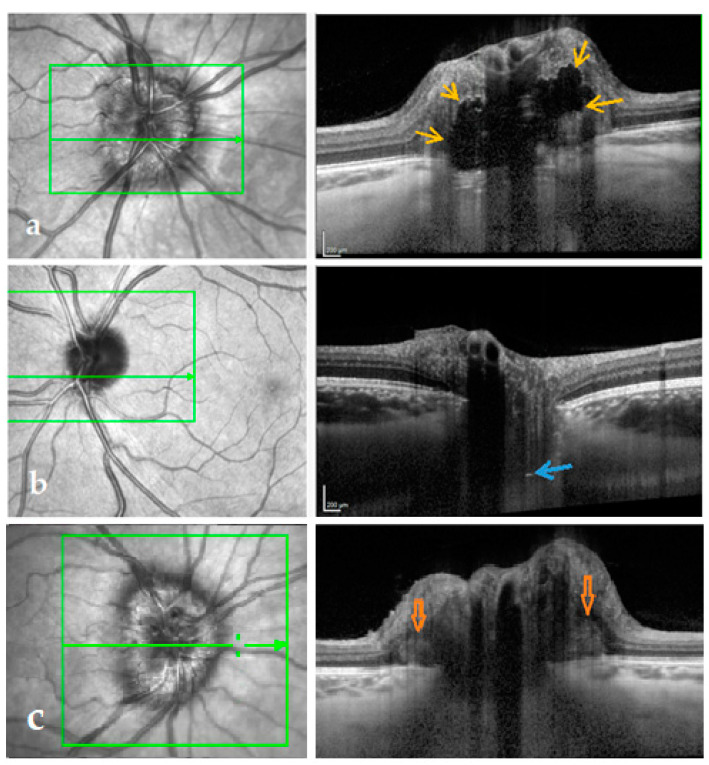
Dense optic nerve head EDI-OCT with corresponding en face OCT imaging. The green line in the green square marks the OCT B-scan. (**a**) IIH patient with ODD (yellow arrows). (**b**) IIH patient with HL (blue arrow). The HL might represent ODD precursors or, in this case, because of the deep location, the signal from the lamina cribrosa. (**c**) IIH patient with PHOMS (orange arrows).

OCT images were analyzed using Heidelberg Eye Explorer (version 1.9.10.0). To minimize the influence of artifacts, HL were defined based on criteria from Malmqvist et al. [10]: (1) located in the prelaminar portion of the optic nerve head, (2) horizontal lines of hyperreflectivity longer than 50 um, and (3) not “broken” by shadowing from overlaying layers. The size was measured using the Heidelberg build in measuring tool. Peripapillary RNFL thickness was determined as the global value from the peripapillary scan. We used the automated values provided by Heidelberg. When necessary, the borders for automatic interpretation were corrected manually. The volume of the GCL was determined as the autogenerated volume from the macular OCT choosing 1-, 3-, and 6-mm EDTRS rings in the dropdown menu in the autogenerated thickness map. The presence of ODD, HL, and PHOMS was based on the ODDS Consortium guidelines for ODD diagnosis on OCT [21]. Briefly, ODD were identified as hyporeflective signal-poor structures with a complete or partial hyperreflective circumference located anterior to the lamina cribrosa, see Figure 1. All scans were thoroughly reviewed by two independent observers (E.W. and L.M.). Clear cases of ODD, HL, and PHOMS were obtained. Both observers were blinded to medical records and each other’s results. In cases with diagnostic discrepancy, an expert ophthalmologist (S.H.) was consulted and final interpretation of the images was made on a consensus basis.

Statistical analyses were performed using the non-parametric Mann-Whitney U Test with a two-tailed hypothesis. *p*-values < 0.05 were considered statistically significant. Data were presented as median values with range.

Patients were divided into 3 groups: Patients with no ODD and/or HL (IIH, no ODD or HL), patients with ODD (IIH + ODD), and patients with HL (IIH + HL). RNFL and GCL were compared in patients with or without HL. Patients with ODD were excluded from this analysis as ODD can affect these parameters. Furthermore, all patients were divided based on the presence of PHOMS (IIH + PHOMS) or not regardless of other findings. The patients with PHOMS were divided into two groups depending on whether they had HL or not (PHOMS, no HL and PHOMS + HL). These groups were compared regarding RNFL and GCL. Data on RNFL and GCL from both eyes were averaged before analysis regardless of uni- or bilateral affection. For example, the RNFL in both eyes of a patient with HL was included even if the patient only had HL in one eye.

At the end of the data collection, we reviewed any available EDI-OCTs of included patients around the time of diagnosis (within 2 days of admission) to find HL and PHOMS. This was done by a single unblinded observer (EW). Results were compared to follow-up data to see whether any HL or PHOMS appeared or disappeared on EDI-OCT between the time points.

## 3. Results

**Figure 2:**
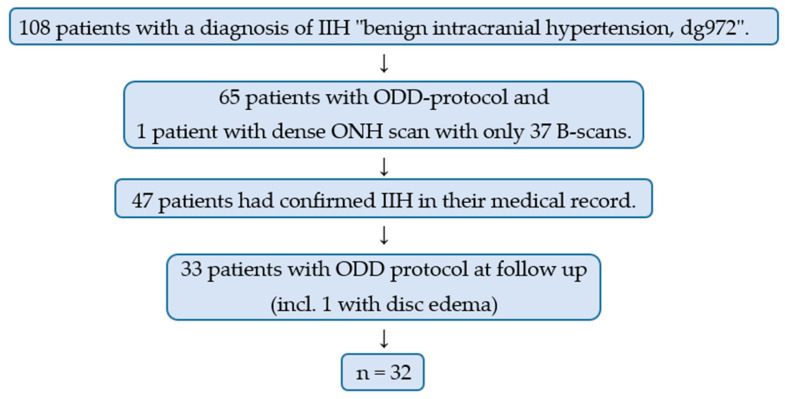
Flowchart demonstrating the inclusion of the 32 patients (64 eyes) in the study. IIH = idiopathic intracranial hypertension; ODD = optic disc drusen; ONH = optic nerve head.

We reviewed the medical records of 108 patients. Sixty-five of these patients had available OCTs according to ODD protocol at any time and one additional patient almost fulfilled the criteria, having 37 B-scans instead of 97 B-scans and was included for subanalysis regarding PHOMS. IIH diagnosis was confirmed in 47 of these patients. Thirty-two patients had ODD protocol available for analysis at the follow-up at least three months after the time of diagnosis. One patient was included even though she only had a follow-up with ODD protocol two months after the time of diagnosis. She had fully resolved disc edema and no remaining subjective or objective symptoms. One patient was not compliant with treatment and was excluded due to too much disc edema at follow-up. OCT interpretation in this patient was unreliable due to shadows from edema. Thirty-two patients (64 eyes) were included. Figure 2. Raw data are available online at www.mdpi.com/xxx/s1 in Table S1.

### 3.1. Age and Gender

The median age was 30 (range 23–66). The study included 30 females and two males. Follow-up varied from two months to seven years. Table 1 presents an overview of patient characteristics.

### 3.2. ODD, HL and PHOMS

**Figure 3:**
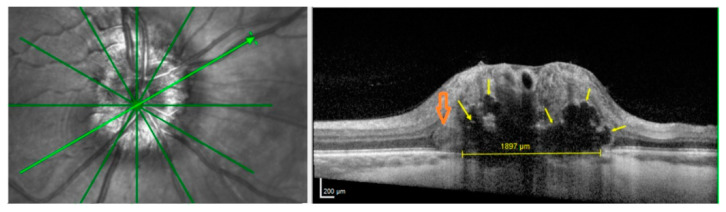
Radial optic nerve head OCT with corresponding en face OCT imaging. The green arrow marks the OCT B-scan. Yellow arrows indicate ODD. Orange arrow indicates a PHOMS. The yellow line in Bruch’s membrane opening indicates scleral canal size. The canal might be overestimated in this B-scan as the ODD possibly hides the edge of Bruch’s membrane to the right.

Of 32 IIH patients, one had ODD (3.1%), 10 patients had HL (31.3%), and 26 patients had PHOMS (81.3%) on EDI-OCT of the optic nerve head at follow up (Table 1). Interrater reliability between the two observers was 93.4% with Cohen’s kappa 0.86, “almost perfect”. The patient with ODD had bilateral drusen, bilateral HL, crowded discs, and bilateral PHOMS, see Figure 3. Nine of the 10 patients with HL had PHOMS, 19 of 22 patients without HL had PHOMS.

We included EDI-OCT performed when papilledema had resolved to ensure sufficient OCT quality. However, 18 patients did have EDI-OCT available around the time of diagnosis (eight of the 10 patients with HL), and it is worth noting that four patients had visible HL in EDI-OCT despite edema at the time of diagnosis (all bilaterally). Four had massive edema and shadows on OCT around the time of diagnosis and presented HL at the follow-up. None of these were resolved at the follow-up. No patients in the group with no HL or ODD presented with HL at the time of diagnosis. Sixteen patients had PHOMS at the time of diagnosis and none had disappeared at the follow-up.

### 3.3. Peripapillary RNFL and Macular GCL

There was no significant difference in peripapillary RNFL or macular GCL comparing patients with HL (the ODD patient was excluded from this analysis) to patients without ODD or HL (*p =* 0.1285 and *p =* 0.1835). Additionally, there was no significant difference in RNFL thickness or GCL volume regarding patients with PHOMS compared to no PHOMS (*p =* 0.32715669 and *p =* 0.27347259) and when comparing patients with PHOMS in addition to HL with PHOMS and no HL (*p* = 0.0854 and *p* = 0.8808).

## 4. Discussion

We found ODD in one (3.1%) of 32 patients with IIH. In comparison, the reported prevalence in the general population is 0.3–2.0% [19]. A recent histopathologic study found an ODD prevalence of 1.8% in enucleated eyes [19]. Considering the diagnostic difficulties concerning ODD the prevalence in this study must be considered rather comparable to the prevalence in the general population.

No pathogenic relationship between IIH and ODD has been shown. In 2016, an ODD prevalence as high as 19% was reported in patients with resolved papilledema from IIH [20]. The authors suggested a noncoincidental relationship. In this study, the ODD diagnosis was placed if the drusen were visible on fundus photos, autofluorescence, B-scan ultrasound, computed tomography scan, or OCT. The EDI function was not employed in the OCT that was used in this study. Instead, the authors relied on the taper of the so-called subretinal hyporeflective space (SHYPS) as a sign of ODD. It has subsequently been determined, that SHYPS was an artifact of early generations of OCT, for a full discussion see [23].

ODD have been related to small scleral canals and crowded optic discs [24,25,26]. One ODD patient in our study did have crowded discs (Figure 3), but due to our small sample size, larger studies are needed to examine anatomical characteristics in IIH patients with ODD compared to IIH patients with no ODD. The prevalence of ODD in this study matches the prevalence of ODD in the general population in favor of a coincidental relationship between IIH and ODD.

HL are believed to possibly represent precursors of ODD [18,21] as the lines are often found in eyes with ODD or in fellow eyes of patients with unilateral ODD [18,21]. In a single study of clinically normal subjects, an HL prevalence of 14.6% has been reported using a comparable protocol for dense optic nerve head EDI-OCT [27].

The present study is the first to systematically investigate HL in patients with resolved papilledema. HL was detected in 31.3% of IIH patients, which is much higher than what was found in normal adults and normal children. The high prevalence of HL in our patients compared to the prevalence in the general population points at a potential role of crowding for the formation of HL. It might even be, that crowding is a prerequisite for the development of HL. This would explain why ODD are almost solely seen in crowded optic nerve heads and it would also fit with the theory, of ODD developing from HL. In congenital crowding, HL potentially develops into ODD, as the crowding is irreversible, but in acquired crowding, such as in papilledema due to IIH, the crowding clears when the papilledema resolves and therefore ‘secondary’ ODD do not develop.

In this study, we included OCT at the follow-up to ensure a high imaging resolution and did not follow the patients with serial EDI-OCTs to track changes. HL can hide in shadows from edema, and this was likely the case in the 4 patients with no HL around the time of diagnosis but with HL at follow-up, as they all had massive edema. If HL represents compressed tissue, then they might resolve as the crowding clears. We did not see this in any of the four patients who were represented with HL at the time of diagnosis. Future studies are needed to reveal any changes in HL post-crowding.

Peripapillary RNFL was not significantly different in patients with HL compared to patients without ODD or HL (*p =* 0.0408). In this analysis the ODD patient was excluded as RNFL in this patient might artificially appear thickened because of ODD. Dai et al. [26] support this finding as they also found no significant difference in peripapillary RNFL thickness in relation to prelaminar hyperreflective lines.

The difference in macular GCL volume between patients with HL and patients without ODD and/or HL was not significant (*p =* 0.4904). HL do not appear to be biomarkers of a worse prognosis in IIH. One might speculate that ODD and potential ODD precursors could cause more severe edema to develop more rapidly with raised ICP as the ODD themselves are space-occupying or crowding, because more frequent crowded discs occur in patients with ODD [24,25,26], in itself would result in a more rapidly developing disc edema. Large ODD could worsen ischemia in small vessels in the optic nerve head during edema and result in earlier damage to the ganglion cell layer [28,29,30]. However, we only included 1 ODD patient and 10 with HL, not nearly enough to test these hypotheses. To reveal any difference in long-term damage in relation to ODD or HL in IIH patients, further and larger studies are needed.

We found PHOMS in 81.3%. PHOMS are probably a result of slowed axoplasmic transport due to axonal crowding and is thought to be a result of the raised intracranial pressure in IIH patients [22]. When pressure is normalized and axoplasmatic flow is restored, PHOMS regress in some patients. We did not see PHOMS disappearing at the follow-up. Our follow-up varied from two months to seven years and all patients were stable at follow-up. If we had had OCTs from one month after the time of diagnosis (still some papilledema in some patients and therefore ongoing congestion) and again after 3–6 months (no congestion), we might have seen PHOMS disappearing in some patients. PHOMS can also be seen in anomalous discs with no other pathology [31,32]. Hence, they might in some cases have been present before the onset of IIH. In this study, the PHOMS is likely a result of slowed axoplasmatic flow in the majority of patients with a prevalence as high as 81.3% supporting above mentioned studies. There was no significant difference in RNFL thickness and GCL volume in patients with PHOMS compared to patients without PHOMS at follow-up.

Our sample size is clearly a limitation, as ODD are rare, and we only discovered one patient with concurrent IIH and ODD. We did not have any OCTs prior to edema and we cannot say with certainty that the HL evolved post-edema. In this study, we included too few patients to stratify the groups with or without HL or PHOMS based on factors such as the severity of edema and disease duration.

Owing to the lack of longitudinal follow-up data, we were not able to track changes in HL. One cannot completely rule out the possibility that we overestimated HL by misinterpreting artifacts and noise as HL. Inter-rater reliability was high in this study. Though high inter-rater reliability is a strength, we would like to mention that detection of HL can be difficult. In this study, we counted patients with HL and not single eyes. The discrepancy when comparing the two observer’s findings in each eye was higher as sometimes one observer would find HL in both eyes but the other only in one eye. Both observers would then categorize the patient as “with HL” with no registered discrepancy. One artifact that might appear as a hyperreflective line is reflections from lamina cribosa. In the majority of patients, this was not an issue as the HL were more anterior than would be expected in the case of reflections from lamina cribosa. Often, one can get an impression of the limits of lamina cribosa on EDI-OCT. Thus, we hope to have avoided this source error to some extend by not including broken lines and what appeared to be lamina cribosa margins as HL. A strength of the study is the thorough and systematic OCT work up to where we believe to have discovered any potential superficial and deep ODD in the included patients. The method allows us to compare results to recent studies on ODD and HL prevalence in the background population [26].

## 5. Conclusions

Optic disc drusen were found in 3.1%, hyperreflective lines in 31.3%, and peripapillary hyperreflective ovoid mass-like structures in 81.3% of all IIH patients at least three months after the time of diagnosis. We found an ODD prevalence in IIH patients similar to the prevalence reported in the general population. ODD are not suspected to develop secondary to IIH or papilledema of any other cause. The high prevalence of HL and PHOMS suggests these structures are caused by some degree of optic disc congestion or axonal stasis. Future studies are needed to conclude what HL represent in acquired congestion.

## Figures and Tables

**Table 1:** Characteristics of the study patients.

	No ODD or HL	ODD	HL	PHOMS	No PHOMS
n (%)	65.6	3.1	31.3	81.3	21.2
Male/female	2/19	0/1	0/10	0/26	2/4
Median age [range] (years)	30 [22–66]	24	31 [27–49]	31 [22–66]	29 [23–49]
Median RNFL [range] (µm)	96 [60–109]	105/92	103 † [55–123]	96 [60–119]	98 ^ [55–123]
Median GCL [range] (mm^3^)	1,07 [0.79–1.34]	1.26/1.22	1.03 †† [0.85–1.35]	1.07 [0.79–1.35]	1.07 ^^ [0.97–0.23]

^†^*p =* 0.1285 and ^††^ *p =* 0.1835 compared to “no ODD or HL”. ^ *p =* 0.3271 and ^^ *p =* 0.2734 compared to “no PHOMS”.

## Data Availability

The data presented in this study are available in [Table S1, Raw Data].

## Supplementary Materials

The following are available online at https://www.mdpi.com/article/10.3390/life11060584/s1, Table S1: Raw Data, IIH patients with available EDI-OCT of the optic nerve head at follow up.
