# Supplementary material for: OCT Based Interpretation of the Optic Nerve Head Anatomy and Prevalence of Optic Disc Drusen in Patients with Idiopathic Intracranial Hypertension (IIH)
Source: Life (Basel). 2021 Jun 19;11(6):584. doi: 10.3390/life11060584 (PMC8234108; doi:10.3390/life11060584)
Supplement: Supplementary file 1 [file life-11-00584-s001.zip › life-1202374-supplementary.pdf]

IIH patients with available EDI-OCT of the optic nerve head at follow up

RNFL (General) OD, µm	OS	GCL (Macular) OD, mm³	OS
102	109	1.34	1.33
104	96	1.10	1.13
98	97	1.02	1.01
100	100	1.05	1.08
88	88	1.13	1.11
103	100	0.93	0.93
103	106	1.15	1.15
90	78	1.03	1.01
98	104	1.09	1.12
80	86	1.07	1.09
92	89	1.14	1.09
87	84	0.98	1.00
71	69	0.97	0.93
60	77	0.83	0.93
88	92	0.90	0.79
96	99	0.97	0.97
95	74	1.12	0.80
70	60	1.12	0.97
84	105	1.02	1.10
104	104	1.07	1.09
98	101	1.07	1.06
104	96	1.02	1.03
82	84	0.97	0.98
104	104	1.00	1.01
107	97	1.16	1.16
55	73	0.97	1.11
123	122	1.23	1.17
117	119	1.16	1.23
112	111	1.22	1.35
93	92	1.00	0.99
101	98	0.85	0.88
105	92	1.26	1.22

Age at onset, years	Age at data collection, years	PHOMS (1 = yes, 0 = no)
22	24	1
24	25	1
21	23	0
28	31	0
48	54	1
42	43	1
27	33	1
28	31	1
22	28	0
24	27	1
45	47	1
23	30	1
64	66	1
29	30	1
38	40	1
33	35	1
26	27	1
23	23	1
30	30	1
27	27	1
21	22	1
25	31	1
31	33	1
32	35	1
26	31	1
25	29	0
45	49	0
26	27	1
35	36	1
26	27	0
28	28	1
21	24	1

Colors and abbriviations

HL: hyperreflective lines

ODD: optic disc drusen

PHOMS: peripapillary hyperreflective ovoid mass-like structures

RNFL: retinal nerve fiber layer (thickness)

GCL: ganglion cell layer (volume)

EDI-OCT: enhanced depht imaging-optical coherence tomography

OD: right eye

OS: left eye
